# Psychological Determinants of Entrepreneurial Success and Life-Satisfaction

**DOI:** 10.1007/s12144-016-9419-1

**Published:** 2016-02-15

**Authors:** Aneta M. Przepiorka

**Affiliations:** Institute of Psychology, The John Paul II Catholic University of Lublin, Al. Racławickie 14, 20-950 Lublin, Poland

**Keywords:** Action orientation, Entrepreneurial success, Goal commitment, Hope, Life satisfaction

## Abstract

The presented study focused on different stages of the entrepreneurial process. The first group comprised those starting a new business and the second group those who have been through the whole process of creating a new business and have now been operating in the market. The general aim of the article was to examine the relationship between action orientation, hope, goal commitment, entrepreneurial success, and life satisfaction, and to determine the role of psychological characteristics (hope, action orientation) in the entrepreneurial process. The hypotheses were tested on a sample of 344 potential entrepreneurs in the prelaunch stage and 127 actual entrepreneurs in the post-launch stage. To analyze these relationships, multiple-group analysis was conducted.

## Introduction

There is an abundance of definitions and approaches concerning entrepreneurship. The definition of entrepreneurship presented in the Merriam-Webster Online Dictionary, widely acknowledged (see Shane 2008), highlights the act of starting up a new business and all the activities related to this process – organizing, managing, and taking risks inherent in the business. Entrepreneurship is the creation of new enterprises, providing goods and services (Shane and Venkataraman 2000), and new employment opportunities.

Starting a business is preceded by the formation of intention, its implementation, collecting sufficient financial resources, and dealing with bureaucracy. The act of starting up a company is a result of thoroughness, long-term determination in action, and the investment of a great amount of energy and time in putting ideas into practice (Gartner et al. 1994); it may take a long time before the company becomes an active participant in the market (Reynolds and Miller 1992). This process can be seen as goal-directed behavior aimed at starting up a business as well as maintaining and developing it in later stages (Laguna 2013). So far in studies on entrepreneurship, the goal-directed behavior approach has been used, but in this approach only the intention to start up a business has usually been examined (cf. Moriano et al. 2012).

The bulk of research shows that entrepreneurs make a great contribution to the economy and society. However, the knowledge about why some people fail to start a new company while others succeed in doing it is still not sufficient (Reynolds et al. 2004). Business startup is a process (Baron 2007; Kessler and Frank 2009; Reynolds et al. 2004) that has its dynamics, order, and requirements. Different stages of the entrepreneurial process consist of various activities that need to be performed and, at each stage, entrepreneurs have various roles to fulfill (e.g., accountant, market researcher, supplies officer, cleaner, assistant) and a variety of duties to perform: from forming a conception of the business, through elaborating a business plan and implementing it, to running the business. As demands change, the role of some characteristics and skills in the entrepreneurial process is not stable and changes over time depending on the entrepreneurs’ experience at the beginning of this process and later on (Baron 2007; Korunka et al. 2010). According to the psychological approach (e.g., Rauch and Frese 2007) the personality approach may be more justifiable in the case of small businesses, where the entrepreneur is the main decision maker and executor. One possible explanation for this is Mischel’s theory of personality (1973) which states that personality traits are more salient in ‘weak’ situations (i.e., in precarious conditions, with unclear requirements, unspecified structure, and incomplete information) as opposed to ‘strong’ situations. This is because the former allow freedom of behavior and interpretation as well as providing few cues.

The main aim of the present study is twofold. Firstly, it examines whether psychological characteristics, such as action-state orientation, hope, and goal commitment (taken from goal theory) are relevant to entrepreneurs’ success and life satisfaction. Secondly, it investigates the role of specific personality characteristics at the beginning of the prelaunch phase and those who have already been running them for some time. The focus of the present study was on the early stage of entrepreneurial activity, when the probability of failure is quite high; it is a stage fraught with adversities, when poor decisions and inadequate resources may have particularly detrimental effects on business startup. Aldrich and Martinez (2001) stress that, due to selection forces, transition from one phase into another might be quite complicated and demanding, and only the daring few reach the finish line. Identifying the psychological characteristics relevant to success at this stage, examining the personalities of those who make progress towards goal achievement, and comparing this initial stage with later ones, may shed more light on success in entrepreneurship. This approach should answer the question why some potential entrepreneurs make progress in launching their businesses and others do not (see Johnson et al. 2006). Moreover, analyzing the psychological characteristics that can function as resources in coping with adversities stems from the practical need to design activities fostering the achievement of entrepreneurial goals in the beginning and later on. The differences between the stages of the entrepreneurial process can be captured by studying individuals who are in the process of creating their businesses and have undertaken activities to that end and comparing them with entrepreneurs who are at a later stage in their activity. This means that, in this article, we will deal with two groups of participants: the first group is those who have taken explicit steps to start a new business and the second group consists of those who have gone through the whole process of creating a new business and have now been operating in the market for some time. Referring to the term used in the subject literature (cf. Laguna 2006, 2008) those individuals from the first group who have taken steps to start a business and are in the phase of doing this, are called *potential entrepreneurs*. They are still in the prelaunch phase, consisting of several tasks to be accomplished, namely: recognizing opportunities, developing an intention to start a business, and accumulating the resources necessary for starting it (Baron 2007). The second group was labeled *entrepreneurs*. The group outside the scope of this study is ‘dreamers’, also called latent entrepreneurs, who only think about being self-employed and whose entire activity remains only in their minds, without the business actually being initiated (Blanchflower 2004; Learned 1992).

Why were these particular personality characteristics chosen? As stated above, in the literature on goal-directed behavior, action-state orientation (e.g., Diefendorff et al. 2000) and hope (e.g., Peterson and Byron 2008; Uy et al. 2009; Youssef and Luthans 2007) influence goal engagement and effectiveness in achieving goals in work and entrepreneurship. According to Kuhl (1994), there are three dimensions of action-state orientation: decision-related action orientation (AOD), failure-related action orientation (AOF), and performance-related action orientation (AOP). Hope reduces the negative effect of a job and correlates positively with work satisfaction and negatively with work burden (Hmieleski and Carr 2007). Individuals with a high level of hope displayed stronger entrepreneurial intention (Jensen and Luthans 2006). Uy et al. (2009) revealed that hope is a mediator between positive affect and effort. Hope influences the value and probability of achieving entrepreneurial goals (Laguna 2008). What is more, hope is related to life satisfaction (e.g., Bailey et al. 2007).

Since broad personality traits are not directly related to business success (cf. Rauch and Frese 2007), goal commitment was introduced as a moderating variable between psychological characteristics and entrepreneurial success. Although researchers stress the role of motivation in the process of achieving goals as a mediator between general psychological characteristics and performance (Locke and Baum 2007), there is still a gap in the current literature on human agency in the process of achieving success that should be acknowledged more robustly. Goal commitment can be an indicator of human motivation (cf. Koo and Fishbach 2008) and is defined as the amount of effort, time, and energy contributed in a long-term perspective to goal achievement while unwilling to decrease the level of goal difficulty (Campion and Lord 1982; Wofford et al. 1992). As a result of a meta-analysis of 60 articles on goal commitment, Klein and colleagues (1999) confirmed that engagement in pursuing a goal enhances the probability of achieving that goal. Being committed to the goal correlated with the final effect of the work (*r* = .23; *p* < .01). Moreover, goal commitment had positive associations with the outcomes of one’s actions and a positive influence on life satisfaction (Emmons 1986; Freund and Baltes 2002). As has been shown, effort is related to progress in the process of starting a company (Renko et al. 2012). However, the degree to which goal commitment interacts with entrepreneurial success has not been considered in the literature.

What are the accurate measures of success at different phases of the entrepreneurial process?

Baron (2007) recommends using different measures to capture goal achievement at different stages of the process. For those who are in the prelaunch phase, progress in company startup and strength of intention may be good indicators of achievement. Another measurement issue was raised by Cohen et al. (2008), who indicated the need to use other indicators besides financial results to evaluate performance. A study by Gorgievski et al. (2011) indicated that, for small business owners, it is not only the objective criteria (e.g., income or turnover) that count and that assessment of entrepreneurial success should be expanded to include personal satisfaction and subjective criteria. What is of note is that personal criteria ranked higher than business criteria.

## The Present Study

In this study, the relationships between psychological characteristics (AOF, AOD, and hope), goal commitment, entrepreneurial success, and life satisfaction were analyzed. Goal commitment was expected to be a mediator in the relationship between psychological characteristics and success.

The following hypotheses were formulated concerning both the prelaunch and the post-launch phases:

- *Hypothesis 1*: AOD (1a) and AOF (1b) will be positively related to the level of entrepreneurial success.
- *Hypothesis 2*: The higher AOD (2a) and AOF (2b) are, the higher the life satisfaction will be.
- *Hypothesis 3*: There will be a positive relationship between hope and entrepreneurial success.
- *Hypothesis 4*: There will be a positive relationship between hope and life satisfaction.
- *Hypothesis 5*: There will be a positive relationship between goal commitment and entrepreneurial success.
- *Hypothesis 6*: There will be a positive relationship between goal commitment and life satisfaction.
- *Hypothesis 7*: AOF (7a) and AOD (7b) will be positively related to goal commitment.
- *Hypothesis 8*: Hope will be positively related to goal commitment.
- *Hypothesis 9*: Entrepreneurial success will be positively related to life satisfaction.

**Fig. 1:**
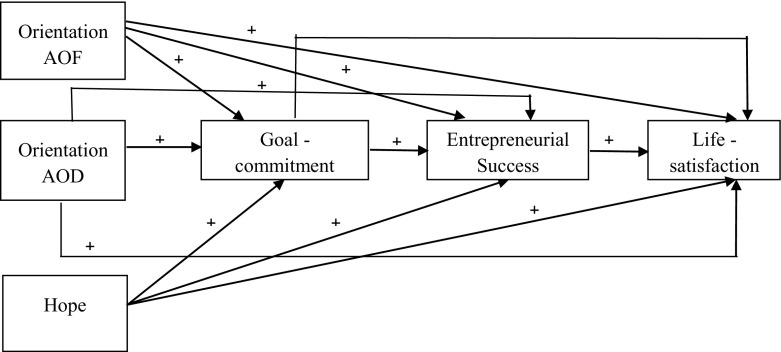
A hypothetical model of relations between psychological characteristics and goal commitment on the one hand and entrepreneurial success and life satisfaction on the other

The variables and the hypothesized relationships between them are included in the proposed model (Fig. 1).

## Method

### Sample

Two groups of subjects took part in the study: potential entrepreneurs and actual entrepreneurs. They came from two voivodeships (provinces) of Poland: Lubelskie and Świętokrzyskie, which are similar with regard to unemployment rate, the number of opened and closed companies, as well as economic and social situation. According to the Polish Agency for Enterprise Development (Raport PARP 2011), at the time the data were collected, there were over 173,000 active businesses in the Lubelskie Voivodeship (which was more than 4 % of all registered businesses in Poland), and over 21,600 new firms were opened there. The number of closed firms amounted to nearly 12,000. The number of microbusinesses was 165,000. In the same period in the Świętokrzyskie Voivodeship there were over 114,000 active firms according to the Polish National Business Registry (constituting less than 3 % of all registered businesses in Poland). A vast majority of them (about 95 %) were small and medium-sized enterprises. In 2010, more than 12,000 firms were opened in that voivodeship, nearly 99 % of which were microbusinesses, and 8142 firms closed down. Some results unrelated to the objective of the current study, have been published elsewhere (Przepiorka 2015).

There are two issues that should be considered while analyzing data to make the study more valuable: that the data on entrepreneurship were collected during the worldwide economic crisis and that the Polish economy remained relatively stable during this period. Moreover, Poland became a free market economy in the early 1990s and knowledge on the current state of entrepreneurship in this country is still scarce, which makes the subject worth researching (cf. Jones et al. 2011).

### Potential Entrepreneurs and Data Collection

Most of the data were collected during seminars designed for people who wanted to start their own companies. The participation criterion was having a business plan accepted by the organizers. Sponsored by the European funds, the seminars ended in choosing the best business plans and granting financial support. The participants were informed about the general goal of the project and received a set of questionnaires in a stamped envelope. They were asked to mail the completed questionnaires to the address provided. The potential entrepreneurs were also recruited during courses in entrepreneurship and economics in the two voivodeships.

The initial pool for the first group comprised 1037 people, whose mean age was *M* = 23 years (*SD* = 4.48). They completed the Entrepreneurial Intention Scale (Laguna 2008; described further in the Measures section), making it possible to select only those who were really interested in starting up a business and had already taken action to launch a company. According to Laguna (2013), the intention to start a business is related to the process of actually starting a business. The participants were instructed to assess their intention to start their company. The scores on this scale ranged from 1 to 5 (*M* = 3.18, *SD* = 0.87). Only the participants with the strongest intention to start a business (over the fourth quartile; *N* = 344, 167 women and 177 men) were included in the final group. Their responses to all the questions on the scale were 4 or 5. This group was labeled *potential entrepreneurs*. Their age ranged from 19 to 52 years (*M* = 23.2 years, *SD* = 4.08). A majority of these people studied and were not employed (73.25 %). They intended to open firms operating in the services sector (26.15 %), in IT (20.64 %), in trade (15.98 %), and in industry (10.52 %).

### Entrepreneurs and Data Collection

The second group comprised entrepreneurs (*N* = 127, 56 women and 71 men). Their age ranged from 22 to 65 years (*M* = 39.14 years, *SD* = 10.34). The small businesses were sought out using the Internet. To be included in the study, businesses had to have been in the market for at least 1 year (the time of their operation in the market ranged from 2 to 20 years, the mean operation time being *M* = 3.93 years, *SD* = 2.73). They also had to employ up to 10 people (*M* = 2.67, *SD* = 2.72), and the owner of each business also had to be its founder. These conditions were crucial for preserving the homogeneity of the groups (cf. Shane and Venkataraman 2000). The data from the group of actual entrepreneurs were collected during individual meetings. After a short telephone interview, an appointment was scheduled. During the meeting, the questionnaires were left for the entrepreneur with a request for him or her to fill them out. In 24.41 % of cases, the business owner was the only employee. For 70 % of the entrepreneurs taking part in the study, the business was their only form of employment, while 23 % were also employed elsewhere. The remaining 7 % did not reply to this question. As regards to the line of business, 47 % of the companies operated in trade, 25 % in construction, 24 % in education and training, and 4 % in production.

### Measures

*Entrepreneurial intention* was measured using the Entrepreneurial Intention Scale (Laguna 2010), a four-item instrument with items scored on a five-point scale ranging from 1 = *not true at all* to 5 = *very true*. This instrument was used only in the group of potential entrepreneurs and contains items such as: *I decided to start my own company* or *As soon as it will be possible, I will open my own company.* Its internal consistency was acceptable (Cronbach’s alpha = .84).

*Action–state orientation* was assessed using the Polish adaptation (Marszał-Wiśniewska 2002) of the Action Control Scale-90 (ACS-90; Kuhl 1994). ACS-90 consists of 36 dichotomous items. High scores on its subscales indicate greater action orientation. Two out of three subscales were used: AOD (e.g., *When I know I must finish something soon*: (a) *I have to push myself to get started*, or (b) *I find it easy to get it over and done with*) and AOF (e.g., *When I am told that my work has been completely unsatisfactory*: a) *I don’t let it bother me for too long*, or b) *I feel paralyzed*). Both subscales had good reliability in the present study (AOF’s alpha = .81 and AOD’s alpha = .76).

*Hope* was assessed using the Polish adaptation (Laguna et al. 2005) of the Adult Dispositional Hope Scale (Snyder et al. 1991). It consists of 12 items: four are distractors, four items concern agency thoughts (e.g., *I energetically pursue my goals*), and four items refer to the pathways of thoughts (e.g., *There are lots of ways around any problem*). Participants respond on an eight-point continuum (1 = *definitely false* to 8 = *definitely true*). The internal consistency was alpha = .88 for the whole scale, alpha = .82 for the agency subscale, and alpha = .81 for the pathways of thinking subscale.

*Goal commitment* was assessed using three subscales from the *Goal Questionnaire* (Zaleski 1991). All items were scored on a seven-point scale. Referring to action in the current project, three subscales measured: *effort* (e.g., *I would rate the intensity of my effort made to accomplish a given goal as*), *perseverance* (e.g., *Despite being tired, I take some action to accomplish a given goal*), and *satisfaction with action* (e.g., *I am satisfied with the actions I undertake toward meeting my goal*). All the subscales had good internal consistency, ranging from alpha = .87 for satisfaction with action to alpha = .95 for effort. On the basis of Anderson and Rubin’s (1949) test, the subscales were grouped into one dimension, labeled Goal Commitment. It explained 67.45 % of variance in the group of entrepreneurs and 92.26 % in the group of potential entrepreneurs. The internal consistency for the Goal Commitment dimension was alpha = .97. The instruction on the Goal Questionnaire was modified to correspond to the phase of the entrepreneurial process. Participants were asked to imagine their goal and to refer to this goal in their responses. For entrepreneurs, the goal was the success of their company and for potential entrepreneurs, it was launching their business.

*Entrepreneurial Succes*s. Different measures of success were used in the groups of potential and actual entrepreneurs because each stage in the entrepreneurial process involves different activities. In the group of potential entrepreneurs, success was measured with the *Scale of Pre-Launch Achievements* (Przepiorka 2011), consisting of 11 items rated on a five-point Likert scale (from 1 = *do not agree at all* to 5 = *agree completely*). The higher the score, the higher the achievement and the more advanced the participant in the process of starting up his or her business (e.g., *I have thoroughly elaborated a business plan; I have an idea and vision for my company*). Internal consistency was alpha = .94. The entire scale loaded on one factor, which explained 40.29 % of variance.

In the group of actual entrepreneurs, success was assessed using the *Scale of Entrepreneurial Success*. They were asked to assess their achievements in comparison with those of other entrepreneurs on the scale developed by Baer and Frese (2003) (e.g., *How successful are you in comparison with your competitors? How successful is your business in comparison to other businesses in the same industry and of about the same size?*). They were also asked to assess the company’s progress during previous years in comparison with two main competitors using 10 questions developed by Wiklund and Shepherd (2005) (e.g., increase of sales, increase of income). A five-point Likert-type scale was used for all questions. All items in these two measures merged into one factor, which explained 39.50 % of variance and had acceptable internal reliability (alpha = .89). These two assessments combined had been used together in previous research by Unger et al. (2008).

*Life satisfaction* was measured with the Polish adaptation of the *Satisfaction With Life Scale* (SWLS; Diener et al. 1985). Example items of this instrument include: *The conditions of my life are excellent* or *I am satisfied with my life*. SWLS consists of five items rated on a seven-point Likert scale (1 = *strongly disagree* to 7 = *strongly agree*). The internal consistency of the scale was alpha = .85.

## Results

### Preliminary Analyses

To test the hypotheses on the relationships between variables (H1–H9), Pearson’s *r* correlations were computed. The intercorrelations between the variables are presented in Tables 1 and 2. In both groups, success correlated positively with psychological characteristics (AOD, AOF, and hope), meaning Hypotheses 1a, 1b, and 3 were supported. Additionally, those who were more action-oriented (AOF, AOD) were more satisfied with their life; the same relationship was found between hope and life satisfaction, confirming Hypotheses 2a, 2b, and 4. Furthermore, those who were more committed to their goals achieved greater success – which confirmed Hypothesis 5. As hypothesized (H6), there was a positive association between goal commitment and life satisfaction. In accordance with Hypotheses 7a, 7b, and 8, goal commitment correlated positively with psychological characteristics (AOF, AOD, hope). Life satisfaction correlated positively with entrepreneurial success, which confirmed Hypothesis 9. The above associations occurred in both groups.

**Table 1:** Means, standard deviations, and correlations between study variables for actual entrepreneurs (*N* = 127)

Variables	M	*SD*	1	2	3	4	5	6
1. AOF	0.49	0.27	-					
2. AOD	0.68	0.23	.51***	-				
3. Hope	6.24	0.98	.45***	.47***	-			
4. Goal commitment	5.57	0.84	.33***	.53***	.43***	-		
5. Entrepreneurial success	3.40	0.60	.34***	.27**	.31***	.46***	-	
6. Life satisfaction	4.69	1.17	.30**	.28**	.44***	.35***	.30***	-

** *p* < .01; *** *p* < .001

**Table 2:** Means, standard deviations, and correlations between study variables among potential entrepreneurs (*N* = 344)

Variables	*M*	*SD*	1	2	3	4	5	6
1. AOF	0.64	0.27	-					
2. AOD	0.74	0.23	.65***	-				
3. Hope	6.19	0.94	.41***	.48***	-			
4. Goal commitment	4.69	1.25	.40***	.33***	.25***	-		
5. Entrepreneurial success	1.82	0.69	.52***	.44**	.24***	.49***	-	
6. Life satisfaction	5.00	1.07	.43***	.46***	.54***	.32***	.35***	-

** *p* < .01; *** *p* < .001

### Comparison Between Groups

To examine whether the relationships between variables were the same in the prelaunch and post-launch phases, multiple-group analysis using the maximum likelihood (ML) technique was computed in AMOS 18.0. This method made it possible to test the data in both groups simultaneously, as well as to verify to what extent the compared groups were similar (Byrne 2010) and whether the same relationships explained the process of achieving success and life satisfaction in potential and actual entrepreneurs. Covariances between variables and the equalities of path coefficients and intercepts across two samples – potential and actual entrepreneurs – were tested and found to be different. All analyses were performed on correlation matrices.

Next, the model’s goodness of fit was assessed in terms of the following indices: chi-squared, root mean square error of approximation (RMSEA), *p*-value for H0 (PCLOSE), goodness of fit index (GFI), adjusted goodness of fit index (AGFI), Tucker-Lewis Index (TLI), and comparative fix index (CFI). Significant chi-squared test, a value of RMSEA lower than .05, a value of PCLOSE higher than .50, CFI equal to or higher than .90, as well as GFI and AGFI higher than .95 are indicative of an acceptable fit (Byrne 2010; Hu and Bentler 1999). The configural (unconstrained) model was taken as reference. The configural model had an acceptable fit to the observed data across two groups. In the next step, structural weights, structural covariances, and structural residuals were constrained as equal across the two groups. To compare the obtained models, the Δχ2 criterion and ΔCFI were used. Non-significant Δχ2 and the difference of ΔCFI not exceeding .01 indicate invariance across groups (Cheung and Rensvold 2002). The comparisons of models are presented in Table 3.

**Table 3:** The results of model comparisons

	Δχ^2^	Δ*df*	*p*	ΔCFI	*χ* ^2^	*df*	*p*	RMSEA	PCLOSE	TLI	CFI	GFI	AGFI
Unconstrained (configural)	-	-	-	-	10.066	6	.122	.038	.635	.977	.995	.993	.951
Measurement constrained	5.575	6	.472	.001	15.641	12	.208	.026	.889	.990	.996	.989	.962
Structural covariances constrained	13.750	12	.317	−.002	23.816	18	.161	.026	.933	.989	.993	.983	.960
Structural residuals constrained	24.345	15	.059	−.01	34.411	21	.033	.037	.824	.978	.985	.975	.950
Structural weights constrained	31.660	9	.000	−.02	41.726	15	.000	.062	.172	.939	.970	.972	.911

The difference between the *χ*2 value for the configural model and the *χ*2 value for the structural weights constrained model was statistically significant. The modification indices showed that the release of constraints in the structural weights would improve the model’s fit to a significant extent. In both groups, the model with restricted covariances, some structural weights, and structural residuals, fitted the data well (*χ* ^2^ = 15.641, *df* = 12, *p* = .208, *χ* ^2^ */df* = 1.303, RMSEA = .026, PCLOSE = .889, TLI *=* .990, CFI *=* .996, GFI = .989, AGFI = .962). The effects (direct, indirect, and total) for the measurement model are presented in Tables 4 and 5.

**Table 4:** Direct, indirect, and total effects for the configural model of relations for entrepreneurs (*N* = 127)

	Goal commitment Entr. success Life satisfaction					
Effects	Unst. est.	St. est.	Unst. est.	St. est.	Unst. est.	St. est.
AOF						
Direct	.67***	.18	.53**	.24	.44*	.10
Indirect	.00	.00	.17	.08	.27	.06
Total	.67***	.18	.70**	.32	.71*	.16
AOD						
Direct	1.44***	.34	−.19	−.07	.00	.00
Indirect	.00	.00	.37	.14	.23	.05
Total	.44***	.34	.18	.07	.23	.05
Hope						
Direct	.20*	.18	.00	*.*00	.47***	.37
Indirect	.00	.00	.05	.08	.04	.03
Total	.20*	.18	.05	.08	.51***	.40
Goal commitment						
Direct	-	-	.25***	.43	.13*	.11
Indirect	-	-	.00	-	.07	.06
Total	-	-	.25	.43	.20*	.17
Entrepreneurial success						
Direct	-	-	-	-	.26***	.13
Indirect	-	-	-	-	.00	.00
Total	-	-	-	-	.26***	.13

*Unst. est.* unstandardized estimate, *St. est.* standardized estimate

*p < .05;** *p* < .01; *** *p* < .001

**Table 5:** Direct, indirect, and total effects for the configural model of relations for potential entrepreneurs (*N* = 341)

	Goal commitment		Entrepreneurial success		Life satisfaction	
Effects	Unst. est.	St. est.	Unst. est.	St. est.	Unst. est.	St. est.
AOF						
Direct	.67***	.23	.67***	.27	.44*	.11
Indirect	.00	.00	.19	.08	.31	.08
Total	.67***	.23	.86***	.35	.75*	.19
AOD						
Direct	.57*	.16	.59***	.20	.00	.00
Indirect	.00	.00	.16	.05	.27	.06
Total	.57*	.16	.75	.25	.27	.06
Hope						
Direct	.07	.08	.00	.00	.47***	.42
Indirect	.00	.00	.02	.03	.01	.01
Total	.07	.08	.02	.02	.48***	.43
Goal commitment						
Direct	-	-	.29***	.34	.13*	.10
Indirect	-	-	.00	.00	.07	.06
Total	-	-	.29	.34	.20*	.16
Entrepreneurial success						
Direct	-	-	-	-	.26***	.16
Indirect	-	-	-	-	.00	.00
Total	-	-	-	-	.26***	.16

*Unst. est.* unstandardized estimate, *St. est.* standardized estimate

*p < .05;** *p* < .01; *** *p* < .001

AOF made a significant contribution to goal commitment and entrepreneurial success in both groups; however, in the group of potential entrepreneurs this influence was greater. In the group of potential entrepreneurs, AOD increased chances for entrepreneurial success. In the group of entrepreneurs, this effect was only indirect. In the group of entrepreneurs, AOD had a significant contribution to commitment. Goal commitment increased the chances for entrepreneurial success in both groups, but this effect was greater in potential entrepreneurs. The path of hope to entrepreneurial success was insignificant in both groups. In the group of entrepreneurs, hope increased goal commitment, whereas in the group of potential entrepreneurs, this effect was insignificant. AOF, hope, and goal commitment increased life satisfaction in both groups. In the group of actual entrepreneurs, the obtained model explained 33 % of the variance in goal commitment, 28 % in entrepreneurial success, and 30 % in life satisfaction. In the group of potential entrepreneurs, the model explained 16 % of the variance in goal commitment, 41 % in entrepreneurial success, and 37 % in life satisfaction.

## Discussion

In this study, by foregrounding psychological characteristics such as action orientation (AOF and AOD), hope, and goal commitment on the one hand and entrepreneurial success and life satisfaction on the other, the relationship between entrepreneur personality and entrepreneurial performance was tested. Additionally, two phases of the entrepreneurial process were compared: the prelaunch phase and the postlaunch phase, when the business had already been in existence for some time.

In accordance with the self-regulation perspective (see Kuhl 1994), support was found for Hypotheses 1a and 1b, which predicted AOF and AOD would be related to higher entrepreneurial success in the prelaunch and post-launch phases. In the entrepreneurial process, effective self-regulation and the ability to cope with negative emotions and omnipresent stress are indispensable (e.g., Baumann et al. 2005). As a result of structural equation modeling, it was revealed that AOD was directly related to entrepreneurial success only in the group of potential entrepreneurs. As indicated (Beswick and Mann 1994), action orientation prevents procrastination, enhances effective planning, and enables action initiation; these skills of effective decision making and thorough planning seem to be more important in the group of potential entrepreneurs. In the beginning of the process of business startup, where the probability of failure and discouragement is very high, it is AOD that activates cognitive and emotional processes connected with goal intention, and may be crucial in deciding to seize the opportunity. Being persistent, characteristic of people exhibiting action orientation, is paramount in the entrepreneurial process (Locke and Baum 2007). Putting the role of action orientation into the broader framework of self-regulation theory (cf. Kuhl 1994), shows that the ability to act effectively while facing a situation of decision making and planning, or a situation of failure, may be important in achieving and maintaining desired progress in goal pursuit. Since physical energy is limited, the way we allocate it is crucial; otherwise conflicting motives may lead to ego depletion (Baumeister and Vohs 2007), resulting in decreased effort and lower performance.

Potential entrepreneurs need efficient self-regulation to facilitate bringing their plans and dreams to fruition and help them, as it were, to cross the Rubicon (cf. Heckhausen and Kuhl 1985), the decisive point of no return. As has been shown (cf. Hechavarria et al. 2012), more thorough planning leads to higher perseverance and better performance in potential entrepreneurs. Entrepreneurs who are action-oriented motivate themselves; they are more persistent and make decisions autonomously (Baumann and Kuhl 2005). These characteristics connected with action orientation may be decisive in how entrepreneurs fit into their work environment and face up to the challenges inevitably embedded in their job, which in turn leads to higher performance (Markman and Baron 2003).

As regards Hypotheses 2a and 2b on the relationship between AOF, AOD, and life satisfaction, predictions were supported only for AOF, which was directly related to life satisfaction in both groups. This association was in accordance with other findings in the literature (e.g., Marszał-Wiśniewska 1999), where action orientation was related to more positive mood and higher satisfaction.

As the results of Pearson correlations showed, there was an association between hope and entrepreneurial success in both groups. Similarly, in the literature, hope, resilience, and optimism were related to positive organizational behaviors such as performance, job satisfaction, work happiness, and organizational commitment (Youssef and Luthans 2007). However, the multiple-group analysis revealed no direct influence of hope on entrepreneurial success. Similar multiple-group analysis used by Laguna (2010) to test models, confirmed that hope did not contribute directly to the probability of achieving success. This result may stem from the fact that hope is related to more distant self-beliefs rather than to other specific characteristics and abilities more directly related to the activity itself (Frese 2007).

According to the hypothesis, in the present study, there was a positive association between hope and life satisfaction. This is in accordance with Bailey et al. (2007) who found hope was a predictor of life satisfaction. Hope belonged to the strengths of the Values-in-Action classification that can enhance life satisfaction (Proyer et al. 2013). As Hmieleski and Carr (2007) showed, hope was a kind of buffer for stressful conditions in entrepreneurial work that may result in a more positive life assessment.

The assumptions concerning the role of goal commitment in the relationship between psychological characteristics and entrepreneurial success have been supported. Other researchers also claim that entrepreneurial motivation is a key factor with great impact on the entrepreneurial process (e.g., Collins et al. 2004; Shane et al. 2012). Action orientation and hope correlated positively with goal commitment. In line with other researchers (cf. Seligman 1990), who claim that involvement in goals is a prerequisite for happiness in life, in this study, goal commitment was related to life satisfaction. Bossong (1994) stressed that, thanks to their engagement, individuals perceive the purpose of their action and, consequently, their anxiety decreases. This finding lends strength to the argument that one’s activity leads to a stable increase in life satisfaction (cf. Lyubomirsky et al. 2005).

This study explicitly showed that entrepreneurial success was related to higher life satisfaction in the group of potential and actual entrepreneurs. People are satisfied with their lives to the degree that they successfully realize their own goals (e.g., Emmons 1986). In the literature, there is robust evidence that confirms the positive relationship between well-being and goal achievement (McGregor and Little 1998; Wiese 2007). Those who have important goals enjoy their life more (Schmuck and Sheldon 2001). This result may also be seen as related to the theory of self-efficacy (Bandura 1982). Entrepreneurs who achieve success are more confident about their competency and, therefore, feel more satisfied with their lives. As the literature on motivation shows, human agency and pursuit of opportunities create the foundation for effective performance and success as the outcome (cf. Frese 2007). This is also consistent with self-determination theory, which assumes the existence of three basic needs — autonomy, competence, and relatedness. They are related to higher performance, life satisfaction, or persistence (Ryan and Deci 2000). Locke and Latham (1990) indicated that the higher the performance, the more positive are the emotions and the higher the satisfaction. The literature indicates that those who are self-employed derive more satisfaction from what they do in comparison with those who work in someone else’s company as employees (Blanchflower and Oswald 1998; Schjoedt 2009). Entrepreneurs are more independent, they have experienced a greater variety of duties, and the results of their effort are more evident. Those factors may result in higher life satisfaction.

The obtained results confirmed that considering goal commitment as a moderator between psychological characteristics and goal achievement is a promising direction for future research on the entrepreneurial process and life satisfaction. The differences between groups indicate that all stages have their own specificity and although they require similar characteristics they play different roles (cf. Baron 2007). The presented model explained more variance in the group of potential entrepreneurs. We may conclude that personality characteristics may be more important at the beginning of this process. However, goal commitment in the group of entrepreneurs was explained better. This may be because the goal is more concrete and specific for them and they are more committed to it as part of their job.

### Practical Implications

The presented results may be of relevance to practitioners who deal with the education and training of potential and actual entrepreneurs. Educators and trainers should include aspects of self-belief and the self-regulatory mechanism in their training courses. As shown in the literature (Laguna 2013), not only developing self-efficacy in entrepreneurs but also hope, motivation, goal commitment, and action in pursuit of success, should be regarded as decisive factors in achieving goals. The importance of will power, reducing negative and ruminative thoughts, may be conducive to starting and running a company efficiently. Entrepreneurs should be taught how to react in cases of adversity and failure as well as how to influence the environment and find an alternative to waiting passively for what may come.

It seems that attribution theory (Heider 1958; Kelley 1967, 1973) would be beneficial in training to explain the role of beliefs about perceived causes of success and failure, their stability, and their locus, and to increase understanding of what and why something happened. This theory and its application should be integrated into teaching programs; it describes how people attribute the causes of events or behavior and, based on their assumptions, interpret the outer world. These attributions impact on perceivers’ subsequent thoughts, emotions, and behaviors. To put it straightforwardly, it is not the outer world, but what entrepreneurs have in their minds that counts more. The importance of planning should also be emphasized. Being proactive has been shown to be a distinguishing feature in potential entrepreneurs (Przepiorka 2010).

The presented findings may also be useful for policymakers and representatives of financial institutions by showing them which direction should be taken to support and stimulate entrepreneurship. The accumulating knowledge on entrepreneurial process, its phases, and challenges each entrepreneur has to face, may help in projecting interventions tailored to the specificity of each phase and prepare entrepreneurs to overcome difficulties. Such interventions may include organizing internships for potential entrepreneurs, or meetings with entrepreneurs as role models, supporting entrepreneurship networks.

### Limitations and Future Research

Several limitations of the study should be acknowledged. First, the research was based on self-assessment. However, the considerable size of the group may have diminished the effect of this kind of procedure. The process of selecting potential entrepreneurs was based on self-reported data but, as indicated in previous research (cf. Laguna 2013), intention is a good predictor of starting up a business, so we may assume that this method is reliable. On the basis of structural equation modeling applied in the study, it is not possible to draw conclusions concerning causal relationships, but by comparing different stages of the entrepreneurial process, we gain new insight into this phenomenon. What is more, the sizes of the two compared groups were not equal, which may also have influenced the results. Yet, to balance these flaws, one advantage of this study is that data were collected directly from entrepreneurs: the people who are starting or about to start their own company have the best knowledge about their intentions, activities, and the whole process (Gatewood et al. 1995). Although the two samples — potential and actual entrepreneurs — had different backgrounds, a similar procedure was successfully applied in other studies (cf. Foo 2011) and showed some significant relationships. The results from the group of entrepreneurs who had been running a company for some time might have been affected by survival bias; on the other hand, they provide information about the psychological factors that distinguish successful entrepreneurs. In both groups there was a slight prevalence of males, but this reflects the general trend (Amorós and Bosma 2014) as this pattern was found in many countries.

Future research should include the type of goals and their perceived value when studying the link between goal setting and entrepreneurial performance. Including value orientation may help explain why entrepreneurs devote time, effort, and crucial resources to achieving specific goals. It would also be valuable to relate goal commitment to company development planning strategy — for instance, growth, innovation, or specialization. The job satisfaction measure, besides life satisfaction, assesses the contribution of satisfaction and specific areas to life satisfaction in general. This study analyzed entrepreneurs’ personality and activity only, but it would also be important to investigate other factors related to entrepreneurial success, such as environment, resources, and interactions with the business startup process (see Kessler and Frank 2009). This would require a more interdisciplinary approach.

## Conclusions

The focus on goal-directed behavior in entrepreneurship presented in this paper represents a new approach to the entrepreneurial process. This empirically verified model elucidates the importance of action orientation, hope, and goal commitment at different stages of the entrepreneurial process. As a result of comparing potential and actual entrepreneurs in terms of these psychological characteristics, some differences were revealed between these two phases. This study also shows that progress in achieving a goal is accompanied by an increase in life satisfaction.
